# Fataluku medicinal ethnobotany and the East Timorese military resistance

**DOI:** 10.1186/1746-4269-3-5

**Published:** 2007-01-22

**Authors:** Sean WM Collins, Xisto Martins, Andrew Mitchell, Awegechew Teshome, John T Arnason

**Affiliations:** 1 Department of Biology, University of Ottawa, Ottawa, Canada; 2 USC Canada-East Timor, Dili, East Timor; 3 Northern Australian Quarantine Strategy, Darwin, Australia

## Abstract

**Background:**

An ethnobotanical study of medicinal and poisonous plants used by the East Timor resistance was undertaken in the Lautem District of East Timor to study medicinal plant use in the region. Interviews were conducted with a single key consultant from the resistance army who belonged to the Fataluku culture. This study is of importance as a historical document and because no previous medicinal ethnobotanical studies on this region exist.

**Methods:**

A rapid ethnobotanical survey of medicinal and poisonous plants was conducted through the proposed Conis Santana National Park in the Lautem district of East Timor. Medicinal and poisonous plants were identified by a Consultant and data was collected by the authors using classical descriptive ethnobotanical techniques (i.e. no quantitative measures) through an unstructured open ended interview.

**Results:**

During the survey 40 medicinal and poisonous plants were identified by the Consultant and collected by the authors. Defining characteristics of the Consultant's knowledge include a high frequency use of trees, heavily forested habitats, leaves, decoctions and drinks for a range of conditions relevant to a resistance army.

**Conclusion:**

Despite limitations of the study, important contributions of this study include preservation of a part of the cultural history of the resistance movement and traditional botanical knowledge of the Fataluku. Furthermore, initial findings may indicate that traditional botanical knowledge is unique amongst different East Timorese cultures in terms of plant selection.

## Background

In May 2002, East Timor emerged as the newest nation in the world after a long and brutal Indonesian occupation which was responsible for the loss of approximately 100, 000 to 200, 000 East Timorese lives [1]. Throughout the Indonesian occupation, the East Timorese resistance, then known as Forças Armadas da Libertação Nacional de Timor Leste (FALANTIL), led an armed struggle against the Indonesian army. During the early years of the occupation, FALANTIL had control of a significant portion of the country but was eventually pushed east into the more remote jungle regions of the Lautem District. A long guerrilla conflict was fought in the forests of Lautem where small groups of FALANTIL forces evaded Indonesian troops for nearly 20 years in some cases.

It was essential during the resistance for all FALANTIL soldiers and supporters to ensure a complete working knowledge of useful forest plants to provide food and traditional medicines. The objective of this paper is to document the historical use of medicinal and poisonous plants by the FALANTIL during the resistance. The information was provided by an elder of the Fataluku people who was responsible for traditional medicine in the East Timorese resistance during the Indonesian occupation.

This research was conducted in the proposed Conis Santana National Park which is located along the south coast of the Lautem District of East Timor. The proposed park is the largest continuous forest block in East Timor and is therefore of critical importance to conservation. East Timor is primarily monsoon forest and savanna although the study area also includes small pockets of evergreen rainforest, semi-evergreen rainforest and moist deciduous forests [2,3].

While this area does represent the largest continuous forest block in East Timor, the area has been disturbed in some places by illegal logging and swidden agriculture [4]. The region is a mosaic of primary and secondary forests and small patches of cleared land. One of the most striking features of the region is the extensive karst topography and the rugged Paichau, meaning 'Pig Head' in the indigenous language Fataluku, Mountains that follow the long axis of the island. Furthermore, the area is also remarkable for the extensive wetlands around the community of Mehara [4]. This wetland region is noteworthy for its extensive swamps and Lake Ira Laluro, East Timor's largest lake.

In 2000, the United Nations Transitional Authority in East Timor (UNTAET) implemented Regulation 2000/19, which declared 15 sites as 'Protected Natural Areas' (PNA) as the first step toward environmental protection in East Timor [5]. Such regulations had not been developed by the occupying Indonesian Government and were therefore a priority. Several PNA were established along the southern coast of Lautem District between Lore, Tutuala and Jaco Island which provided the platform for seeking national park status for the region [5]. In September 2003, the Ministry of Agriculture, Forestry and Fisheries of East Timor in collaboration with New South Wales Parks and Wildlife Service and Bird Life International of Australia, submitted a proposal to protect a large area of continuous forest that includes Jaco Island and a swath of land between Lake Ira Laluro and Tutuala, and the mentioned PNA [6]. Several communities are within the proposed protected area's borders and include Tutuala, Mehara and Maupitine. Full national park status is now being sought for the area by the East Timorese Ministry for Agriculture, Forestry and Fisheries with assistance from the New South Wales Parks and Wildlife Service and Bird Life International, with Conis Santana National Park being the proposed name.

Extensive research has been undertaken to document traditional use of medicinal plants throughout the Malay Archipelago [7-11]. However, due to the political instability of East Timor over the last three decades, there has been little biological research of any kind in the country. While preliminary botanical inventories [4] and bird surveys [12] have been completed in the study area, literature concerning the use of medicinal and poisonous plants in the region is absent.

The recent trend in ethnobotany has been a shift from descriptive accounts of plant use by indigenous people to hypothesis testing. This was made possible through the development of consensus indices such as those created by Phillips [13,14] and Trotter and Logan [15]. A limitation of the present report is that consensus indices and hypothesis testing were not possible as only one traditional medicine expert from the resistance army was made available by local authorities. While this report does not utilize quantitative methods, it is a relevant contribution to ethnobotanical literature because the research 1) is undertaken in a biodiversity hotspot relative to other habitats in East Timor, 2) describes medicinal plant use in a conflict situation and 3) aids in the preservation of the unstudied traditional botanical knowledge of East Timor where local conditions continue to make such studies challenging.

## Methods

### General methods

The study consisted of a field trip through the proposed Conis Santana National Park from March 18 – 26, 2004. Former FALANTIL soldiers were hired as route finders through the forest, as specified trails were non-existent. The Consultant for the study was selected by the Chief of a nearby village on the basis that he was the most knowledgeable member of the community concerning the use of medicinal and poisonous plants. During unstructured interviews, medicinal and poisonous plants were identified by the Consultant and collected by the authors. Local name, plant part used, method of preparation and specific uses were all provided by the Consultant and recorded by the authors. The medical conditions treated by the medicinal plants were categorized by the authors according to the method developed by Cook [16].

### Voucher specimens

Voucher specimens of all mentioned plants were collected by the authors in triplicate and stored in 70% alcohol and later dried. One set of vouchers is stored at each of the Universidade Nacional Timor Lorosae'e, Dili, East Timor; the Northern Territory Herbarium, Darwin, Australia; and the University of Ottawa, Ottawa, Canada. Identifications were made by Andrew Mitchell of Northern Australian Quarantine Strategy. Taxonomic identifications were difficult to determine as most samples were sterile.

### The consultant

The Consultant was between 60 and 70 years of age and spoke both Bahassa Indonesian and the local language Fataluku. His spirituality was animist. As is the case with most East Timorese living in rural areas, he was a peasant farmer whose most important crops were corn and cassava. It is important to note that both corn and cassava both originate from the New World. This demonstrates a global exchange of ethnobotanical knowledge which impacts all cultures and findings of ethnobotanical investigation including the findings of this research. While the Consultant's home village was a remote community in the area of the proposed park, his community had been relocated by the Indonesian Government to a roadside location, during the 1970s.

As a youth, the Consultant had been trained by a Fataluku elder from the Consultant's home village in medicinal and traditional plant use. Although the training was informal, the Consultant became an apprentice of the elder and was required to prepare a written account of the knowledge in Bahassa Indonesian. At the present time, the Consultant's teacher lives by himself in the community's traditional territory within the proposed park, residing in limestone caves and surviving by hunting and gathering in the forest and swidden agriculture.

### Ethical approval

Prior to undertaking field activities, the District Administrator for Lautem District was consulted and asked for permission to work in the research area. Furthermore, a meeting was held to explain the objectives of the research and receive consent from the village Chief and elders. Ethical approval was granted by the University of Ottawa Ethics Committee with the understanding that the Consultant's identity would be kept confidential. The Ministry of Agriculture, Forestry and Fisheries of East Timor was consulted prior to conducting research, however, collecting permits were not issued as regulations have yet to be developed. Furthermore, the Consultant explicitly stated that he felt it important to publish his knowledge of medicinal plants to a broad audience.

## Results and discussion

In total 40 plant species were identified by the Consultant and collected by the authors of which 35 were used to treat human ailments, 2 were used for veterinary purposes and 3 were poisons (Table 1). 20 of the medicinal plants were identified to species, 13 to genus and 3 to family and 4 were not identified to any level. Identifications were difficult as many plant vouchers lacked flowers or fruit. The most commonly mentioned families were the Fabaceae (4 species) and the Euphorbiaceae (3 species). When compared to other ethnobotanical studies, the number of medicinal plants mentioned is exceptionally high. For example, Giday *et al*. [17] described the medicinal use of 33 species used by 17 key informants of the Zay people of Ethiopia. Long and Li [18] documented the use of medicinal plants by the Red headed Yao people of China where they listed only 66 medicinal plant species used by healers, herbalist and elders in seven districts.

**Table 1:** Family, botanical name, Fataluku name and specific uses of 40 medicinal and poisonous plant species collected in the proposed Conis Santana National Park.

**Family**	**Botanical Name**	**Voucher**	**Fataluku Name**	**Specific Uses**
Anacardiaceae	*Mangifera indica *L.	UO 19564	Payahi	wounds
Apocynaceae	*Alstonia *species B **R.Br**.	UO 19575	Wiahara	diarrhea
	*Cerbera manghas *L.	UO 19591	Amibya	lactation stimulant
Araceae	*Monstera *species **Adans**.	UO 19570	Nae nae rasa	wounds
Araliaceae	*Schefflera *species B **J.R.Forst. & G.Forst**.	UO 19590	Latuporo	veterinary medicine
	*Schefflera *species C **J.R.Forst. & G.Forst**.	UO 19592	Tufutu	fractures
Arecaceae	*Cocos nucifera *L.	UO 19565	Vata mimiraka	fractures
Caricaceae	*Carica papaya *L.	UO 19607	Muu	malaria
Crassulaceae	*Bryophyllum *species **Salisb**.	UO 19596	Pipivalikerekere	ear infections
Cucurbitaceae	*Momordica *species L.	UO 19604	Kapinu	malaria
Cycadaceae	*Cycas *species L.	UO 19573	Beku	wounds
Dioscoreaceae	*Dioscorea bulbifera *L.	UO 19579	Churailahoo	wild game poison
Euphorbiaceae	*Aleurites moluccana ***(L.) Willd**.	UO 19568	Pokuru	post partum bleeding, internal bleeding
	*Euphorbia atoto ***G.Forst**.	UO 19599	Foy hasa reku reku	low quality breast milk
	*Jatropha curcas *L.	UO 19598	Mutu mutu mimi raka	urinary tract infection
Fabaceae	*Albizia lebbeck *(L.) Benth.	UO 19585	Iparakuliku	pink eye
	*Derris *species B Lour.	UO 19603	Cha	fish poison
	*Pterocarpus indicus *Willd.	UO 19571	Makari	mouth sores
	*Tamarindus indica *L.	UO 19589	Kaylemu	sore joints
Lamiaceae	*Leucas *species **R.Br**.	UO 19587	Muka muka	sores, eye trauma
Lecythidaceae	*Barringtonia asiatica ***(L.) Kurz**	UO 19594	Coru	inflammation
Leeaceae	*Leea indica ***Merr**.	UO 19562	Motiir	diarrhea
Loranthaceae	*Amyema *species B Tiegh002E	UO 19601	Laki soru aku	Urinary tract infections
Malvaceae	*Hibiscus *species *L*.	UO 19563	Varu	Wounds
Meliaceae	Unknown	UO 19580	Pepuru	veterinary medicine
	*Azadirachta indica *A. Juss.	UO 19582	Erua	sore joints
Menispermaceae	*Tinospora smilacina *Benth.	UO 19602	Shururu	lice infestation, snake bite
Myristicaceae	Unknown	UO 19583	Paunete	fish poison
Piperaceae	*Piper *species L.	UO 19584	Tarukukurisa	itching
Poaceae	Unknown	UO 19574	Severoo	weight loss
	*Imperata cylindrica ***(L.) P.Beauv**.	UO 19576	Beerasa	helminth worm infection
Polypodiaceae	*Drynaria quercifolia ***(L.) J. Sm**.	UO 19578	Sa-pu	throat infections
Rubiaceae	*Pavetta *species L.	UO 19561	Lalamoo	tuberculosis
	*Nauclea orientalis ***(L.) L**.	UO 19566	Savele	pink eye, post partum bleeding, internal bleeding
Rutaceae	*Citrus hystrix *DC.	UO 19595	Ami churuku	hepatitis, inflammation
Verbenaceae	*Gmelina philippensis *Lam.	UO 19588	Kapuasamaru	hepatitis
unknown	Unknown	UO 19577	Ai-anu	muscle relaxant
unknown	Unknown	UO 19572	Luluparoo	wounds
unknown	Unknown	UO 19600	Pia pia	dysentery
unknown	Unknown	UO 19567	Tua tua hikari	cough

**Figure 1:**
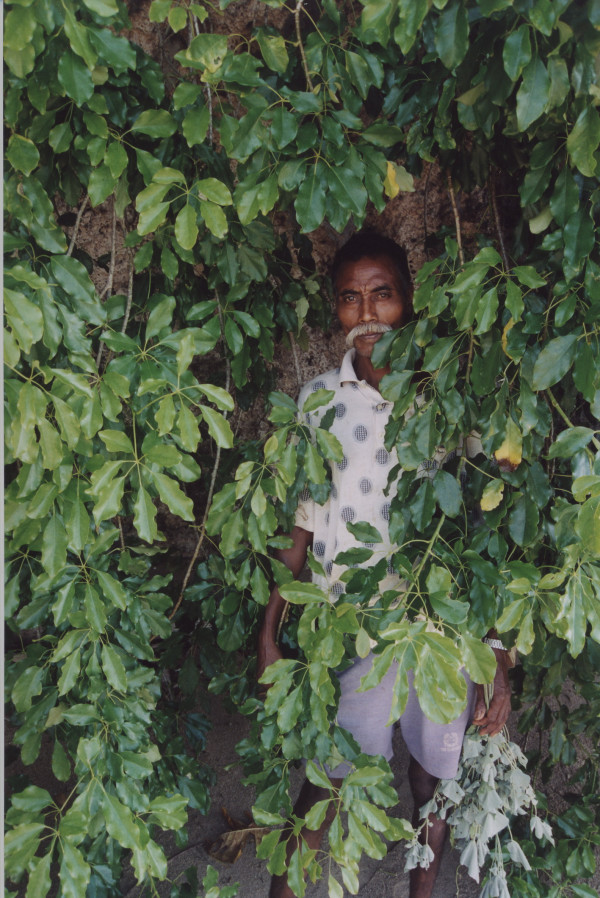
A poultice made from the bark of *Schefflera *species B is used to treat bone fractures.

The most commonly mentioned usage categories were Infections/Infestations, Injuries and Muscular/Skeletal System Disorders (Table 2). The high number of mentions of both Injuries and Muscular/Skeletal System Disorders is a reflection of the Consultant's accumulated experience treating FALANTIL soldiers and East Timorese civilians wounded during altercations with the Indonesian army and militias. While on the field trip, we met a family who had homesteaded within the park. During the Indonesian occupation, the father was returning home along a jungle path from the district capital, Los Palos. The man was ambushed by a group of Indonesian soldiers patrolling the area and was left for dead after a vicious machete attack. Fortunately, the man made it back to his homestead where his wife was able to treat him using traditional medicine. Incidents such as this commonly resulted in serious life threatening wounds such as severe blood loss and fractures. Treatments used include those which act as coagulants and remedies to mend bone fractures. An unidentified vine, used to treat wounds that are bleeding heavily was cut into a foot long section and sap was extruded onto the wound by blowing into one of the cut ends. Poultices were made from the fruit of coconut (*Cocos nucifera*) and from the bark of *Schefflera *sp. B (Figure 1) and applied to broken bones before being secured firmly in place with cloth.

**Table 2:** Frequency of usage category mentions for medicinal and poisonous plant species collected in the proposed Conis Santana National Park.

**Usage Category**	**Number of Mentions**
Infections/Infestations	11
Injuries	7
Muscular-Skeletal System Disorders	5
Poisons	3
Digestive System Disorders	3
Preganancy/Birth/Puerperium Disorders/Effects	3
Skin/Subcutaneous Cellular Tissue Disorders	3
Genitourinary System Disorders	2
Inflammations	2
Veterinary Medicines	2
Culture Bound Syndromes	1
Nutritional Disorders	1
Poisoning Disorders	1
Respiratory System Disorders	1

Of the Infections/Infestations usage category, malaria, hepatitis and pink eye were the most commonly mentioned specific uses. The Consultant noted that malaria is an easily curable disease whose treatment is very common knowledge in East Timor. Papaya (*Carica papaya*) leaves or the leaves of *Momordica *sp. were boiled in water and the decoction drank or used as a shower and drank in the respective remedies. The small intestines of the cuscus (*Phalanger *sp.), a small nocturnal marsupial, were also used to treat malaria. While collecting medicinal plants, the two FALANTIL guides constantly scanned dead standing trees for signs of the cuscus' nest. When a nest was spotted, one of the men climbed the large dead standing trees unaided and removed the cuscus by the tail. The primary reason for hunting the cuscus was for the meat, however, the small intestines can be boiled or roasted and eaten to treat malaria. It was believed that the small intestine has curative properties because the cuscus feeds heavily on *Derris *sp. and *Tinospora smilacina*. *T. smilacina *is used traditionally in Australia to treat infections and inflammatory conditions and was found to have significant anti-inflammatory activity by Li *et al*. [19,20]. Furthermore, *Derris *spp. are traditionally used as fish poisons throughout South East Asia and have highly active compounds known as rotenones [21].

The two veterinary medicines, *Schefflera *sp.A and the unidentified Meliaceae species were respectively used to treat goats and dogs with mange, and also as a general tonic cure for sick livestock. The root of *Dioscorea bulbifera *was either cooked or used raw to poison wild animals such as pigs and dogs. The root was combined with food such as rice or cassava and then left on game trails. Once dead, the meat of the game animal is edible, however, it was mentioned that the toxins accumulate in the organs making them inedible. In the case of the unidentified Myristicaceae and *Derris *sp. the root and bark or stem only were used in the respective preparations. The macerated plant material was added to small confined bodies of water such as tidal pools or creek pools in the dry season. These preparations are effective poisons for shrimp and fish such as eels.

**Figure 2:**
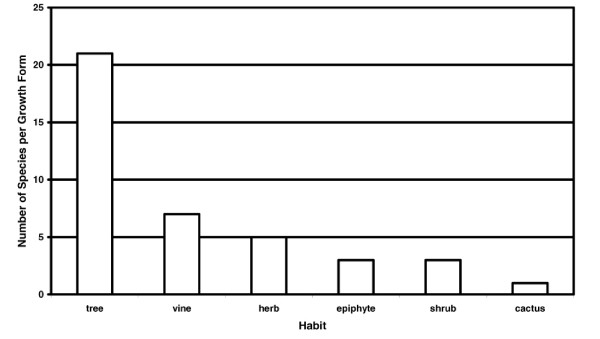
Number of species per growth form for 40 medicinal and poisonous plant species collected in the proposed Conis Santana National Park.

**Figure 3:**
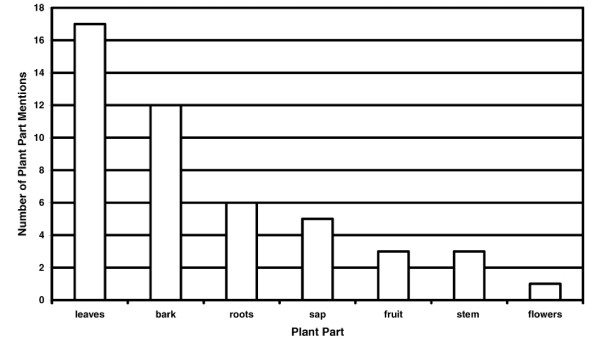
Number of plant part mentions for 40 medicinal and poisonous plant species collected in the proposed Conis Santana National Park.

The plants were analyzed according to frequency of use with respect to plant habitat and growth form, plant part used, method of remedy preparation and route of administration. However, it is important to note that number of mentions for plant part used, method of preparation and route of administration do not equal the total number of species as there were often multiple mentions in these categories per species. Of the 40 useful plant species, 21 were trees while vines and herbs were both used in over 10% of the mentions (Figure 2). Heavily forested areas were mentioned often (35 of 40 mentions) while cultivated fields, beaches and dry steam beds accounted respectively for 2, 2 and 1 of 40 mentions respectively. Leaves (17 of 47) and bark (12 of 47) were the most commonly used plant part (Figure 3). When the bark is used, the outer tissue is removed and the inner bark is collected and prepared. The bark is commonly taken from the tree in long strips and then dried in the sun thereby preserving the remedy for future use.

**Figure 4:**
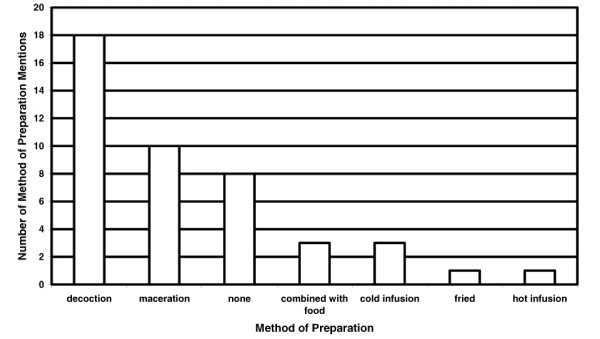
Number of method of preparation mentions for 40 medicinal and poisonous plant species collected in the proposed Conis Santana National Park.

**Figure 5:**
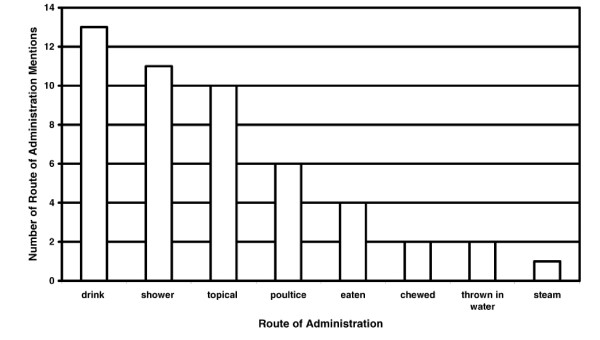
Number of route of administration mentions for 40 medicinal and poisonous plant species collected in the proposed Conis Santana National Park.

Decoctions were used for 18 of the 44 preparation mentions (Figure 4). Macerating (10 of 44) the plant material with a stone or using a traditional mill was also common. No preparation was required for 8 of the uses which were usually sap remedies administered topically or treatments that were chewed or eaten. The most common route of administration was a drink which was used for 13 of the 49 administration mentions (Figure 5). Other oral routes include eating (4 of 49) or chewing (2 of 49). The most common external administration was showering (11 of 49) but topical routes (10 of 49) and poultices (6 of 49) were also used. The only example of combining medicinal plants in a remedy was that of adding the bark from *Aleurites moluccana *to that of *Nauclea orientalis *to treat internal and post partum bleeding.

**Figure 6:**
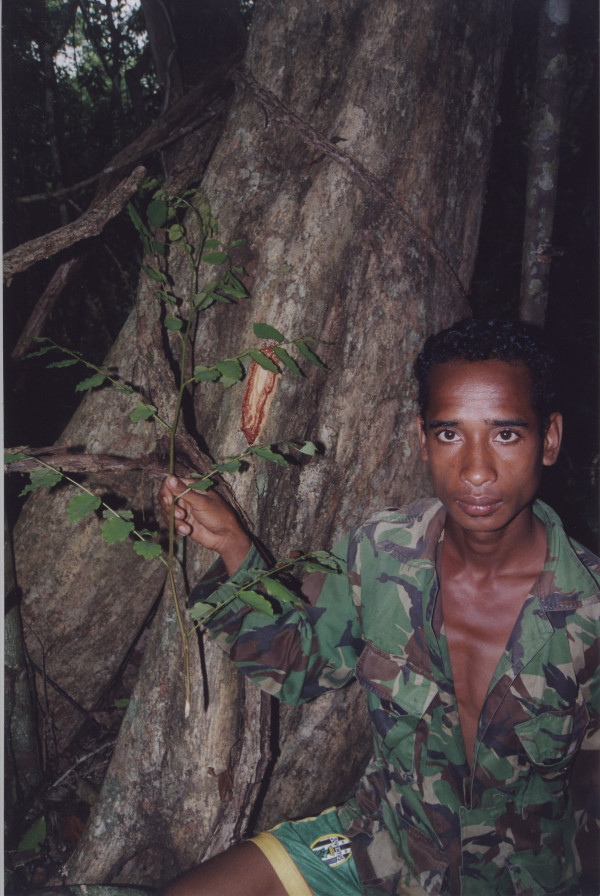
The sap of *Pterocarpus indicus *is used topically to treat mouth sores.

Several of the plants mentioned by the Consultant have been used by other cultures as medicinal plants and have also been investigated for pharmacological activity. For example, *Pterocarpus indicus *(Figure 6) is used to treat "sores and minor wounds" in an unspecified South-East Asian country as cited by Khan and Omoloso [22] and was found to have significant antibacterial activity [22]. *Dioscorea bulbifera *is a traditional Chinese remedy for sore throat and also for tuberculosis as mentioned by Gao *et al*. [23] who found that extracts had anti-tumor effects [23].

The plant species identified by the Consultant are markedly different from the medicinal flora of the Idate and Laklei East Timorese cultures documented in previous ethnobotanical research. Of the 40 plants collected, 32 were different species from the medicinal flora of the Laklei or the Idate people of East Timor consisting of 86 plant species [24]. The Consultant's differing pharmacopeia is most likely due to differing site conditions and perhaps unique cultures. Conditions at Conis Santana National Park include 1) high annual rainfall, 2) low human presence and therefore human disturbance, 3) and large patches of primary and extensive stands of secondary forest exist compared to the heavily degraded landscape that dominates much of East Timor [25]. All of these defining characteristics contribute to significant differences in the flora of Conis Santana National Park and therefore the medicinal plants collected. Furthermore, different cultural groups may have significantly different medicinal plant traditions where the plant species used will be unique as noted by Collins [24]. However, because only one consultant was interviewed in this study it is unclear whether the Consultant's knowledge represents his own specilalized knowledge or the Fataluku medicinal plant tradition as a whole. Furthermore, several of the plants identified by the Consultant are introduced species therefore the suite of plants identified may not represent an ancient Fataluku tradition but a more contemporary body of knowledge subject to a more modern ethnobotanical condition; namely the greater exchange of ethnobotanical knowledge on a global scale. While the medicinal plant species and habitat of collection differ from other areas in East Timor, several similarities exist. For example, the most frequently used plant part (leaves and bark), preparation method (decoctions, macerations and no preparation), habitat (forest) and route of administration (drink) were the same for the Idate, Laklei and Fataluku people [24]. These similarities may indicate unifying characteristics of the East Timorese medicinal plant tradition in general.

The Consultant's knowledge of medicinal and poisonous plants may in part be a result of the degree to which he had to rely on the local flora during the recent political unrest. It was common for entire villages to flee to the mountains to seek refuge from the brutality that began when the Indonesian army invaded in 1975 and continued through to the violence that followed the referendum in August of 1999. Many East Timorese can recount stories of having to survive in the forest under harsh conditions, where they were forced to live on papaya leaves for months at a time as it was too dangerous to return to roadside communities where Indonesian forces had control. Traditional medicine was relied upon heavily to treat ailments since seeking medical attention in city centres such as Dili and Baucau was not an option as these were the strongholds of the Indonesian occupation force. The reliance on the natural environment by FALANTIL soldiers was even greater as they were actively pursued by Indonesian forces in the forest. They could not carry adequate equipment, and as one former FALANTIL soldier stated: "During an ambush, you only had time to grab your gun and flee".

Many East Timorese have a working knowledge of useful plants, however the knowledge of the Consultant is exceptional for two key reasons. First, he had a mentor who instructed him on useful plants and their various preparations. Secondly, he grew up and later lived in close proximity to the stronghold of the East Timorese resistance army, FALANTIL. The Consultant was not a FALANTIL soldier himself, but provided critical support to the resistance by caring for sick soldiers and passing on his knowledge of medicinal plants.

While making the plant collection and conducting interviews, it was apparent that the two former FALANTIL soldiers also knew some remedies by the fact that they were able to clarify certain details. However, younger members of the expedition clearly had little or no knowledge of the plants. This presents a serious obstacle to maintaining traditional medicinal plant knowledge. Fostering interest in medicinal plants among younger members of the community is made difficult by the hospital in the district capital of Los Palos. The pharmaceutical drugs and western treatments offered by the hospital are rendering knowledge of local medicinal plants and their uses obsolete according to the youth. The loss of traditional botanical knowledge is a global phenomenon [26,27] which erodes local and our global cultural heritage.

## Conclusion

The study is a preliminary investigation of the use of medicinal and poisonous plants in a region of the world where, until now, such investigation has been absent. While significant limitations of this study exist, namely a sample size of one and incomplete identification of voucher specimens, initial findings are important as they provide baseline data on traditional botanical knowledge in the area which aids in preserving this type of information for future generations of East Timorese and comparison to future more in depth ethnobotanical study in the area. Although the one key Consultant's knowledge does not describe Fataluku traditional botanical knowledge in its' entirety, the medicinal and poisonous plants used by the Consultant are dramatically different than consultants from other East Timorese cultures, namely the Idate and Laklei people. In fact only 32 of the 40 plants identified in this study are used by either the Idate or Laklei people.

## Competing interests

The authors declare that they have no competing interests.

## Authors' contributions

SC carried out field work, collected and analysed the primary data and drafted the manuscript. XM coordinated field work and logistics and made revisions to the manuscript. AM identified plant vouchers. AT coordinated field work between collaborators in East Timor and Canada and also commented on the manuscript. JTA coordinated field work and made revisions to the manuscript.
